# Network attributes underlying intellectual giftedness in the developing brain

**DOI:** 10.1038/s41598-017-11593-3

**Published:** 2017-09-12

**Authors:** Jiyoung Ma, Hee Jin Kang, Jung Yoon Kim, Hyeonseok S. Jeong, Jooyeon Jamie Im, Eun Namgung, Myeong Ju Kim, Suji Lee, Tammy D. Kim, Jin Kyoung Oh, Yong-An Chung, In Kyoon Lyoo, Soo Mee Lim, Sujung Yoon

**Affiliations:** 1 Ewha Brain Institute, Ewha Womans University, Seoul, South Korea; 2 Interdisciplinary Program in Neuroscience, College of Natural Sciences, Seoul National University, Seoul, South Korea; 3 Department of Brain and Cognitive Sciences, Ewha Womans University, Seoul, South Korea; 4 Department of Radiology, Incheon St. Mary’s Hospital, College of Medicine, The Catholic University of Korea, Seoul, South Korea; 5 College of Pharmacy, Graduate School of Pharmaceutical Sciences, Ewha Womans University, Seoul, South Korea; 6 Department of Radiology, Ewha Womans University College of Medicine, Seoul, South Korea

## Abstract

Brain network is organized to maximize the efficiency of both segregated and integrated information processing that may be related to human intelligence. However, there have been surprisingly few studies that focus on the topological characteristics of brain network underlying extremely high intelligence that is intellectual giftedness, particularly in adolescents. Here, we examined the network topology in 25 adolescents with superior intelligence (SI-Adol), 25 adolescents with average intelligence (AI-Adol), and 27 young adults with AI (AI-Adult). We found that SI-Adol had network topological properties of high global efficiency as well as high clustering with a low wiring cost, relative to AI-Adol. However, contrary to the suggested role that brain hub regions play in general intelligence, the network efficiency of rich club connection matrix, which represents connections among brain hubs, was low in SI-Adol in comparison to AI-Adol. Rather, a higher level of local connection density was observed in SI-Adol than in AI-Adol. The highly intelligent brain may not follow this efficient yet somewhat stereotypical process of information integration entirely. Taken together, our results suggest that a highly intelligent brain may communicate more extensively, while being less dependent on rich club communications during adolescence.

## Introduction

Intelligence is known as the general cognitive capability to reason, plan, solve problem, appraise, and learn quickly from experience^1^. Moreover, intelligence is strongly associated with behavioral patterns and important life outcomes, including health and longevity^2^.

Since the earlier research which investigated the correlation between brain size and intelligence^3^, considerable efforts have been made to identify the neural basis underlying inter-individual differences in intelligence^4–7^. In the early days of research, the alterations in the fronto-parietal gray matter regions as an indexing marker for intelligence have attracted much attention, due to their implicated role in information processing^8^. However, as a growing number of studies have examined white matter connections and network metrics, integrative roles of the brain in networking multiple cortical and subcortical regions have become an increasing interest in investigating intelligence^4^. Recent evidence indicated that inter-individual differences in intelligence may be attributed to the global efficiency and strength of white matter connectivity network across the brain regions^9–11^. Furthermore, the relationships between intellectual ability and functional efficiency of brain network were more pronounced in the frontal and parietal regions^12–14^. Interestingly, the fronto-parietal regions along with the subcortical structures including the thalamus, putamen, and hippocampus are considered as highly inter-connected brain hubs, also referred to as “rich clubs”^5^. Connections between these rich clubs play a key role in promoting efficient information flow in the brain network, despite the high cost of white matter wiring^15–17^. Moreover, global efficiency of the brain network could be influenced by damages to the connections between rich clubs^15^. Therefore, rich club connections can be presumed to optimize global communication efficiency, thus improving global cognitive functions including intelligence^16^. Likewise, a link between the strength of rich club connections and performance on visuo-spatial motor processing was observed in typically developing preadolescent children^11^.

“High intelligence” or “intellectual giftedness” is often recognized from an early age and is characterized as a unique set of highly developed cognitive capacity leading to extraordinary accomplishment^18^. Since the first histological work on Albert Einstein’s brain^19^, a series of studies have examined his postmortem brain in an attempt to identify brain properties that are associated with high intelligence^20–22^. However, one of the most striking findings from these studies was that extraordinary cognitive capability may not be accounted for by the specific structural features of the brain. On the other hand, a few studies using *in vivo* brain imaging have provided preliminary evidence for the neural correlates of high intelligence. Such studies have found that increased functional involvements in the fronto-parietal regions during task performance may be associated with high intelligence^23, 24^. However, there have been surprisingly few studies that focus on the neural basis of ‘high intelligence’ in the context of brain network perspectives.

In this study, we characterized global network attributes for high intelligence, including the role of rich club regions and their connections using the construction of white matter structural network. This study aims to examine whether high intelligence can be attributed to a unique “genius brain” with distinctive network connections or to a “normal brain” with outstanding measurements of intelligence within a normal distribution spectrum. For this purpose, we compared adolescents with superior intelligence to adolescents and young adults with average intelligence. Superior intelligence was classified by intellectual scores that were more than two standard deviations above the average scores^25^.

## Results

The current study included adolescents who had intelligence quotient (IQ) of 130 and higher and who were recruited from high schools for intellectually gifted students in South Korea (n = 25, hereafter referred to as “adolescents with superior intelligence [SI-Adol]”). Twenty-five students with IQ < 120 were recruited from regular high schools as “adolescents with average intelligence (AI-Adol)”. Young adults between ages 20 and 30, with an IQ < 120 (n = 27), were recruited as “young adults with average intelligence (AI-Adult)”. Characteristics of participants are presented in Table 1.

**Table 1:** Characteristics of study participants.

	Adolescents with SI (n = 25)	Adolescents with AI (n = 25)	Young adults with AI (n = 27)
Age — yr	17.0 ± 0.9	17.0 ± 0.8	25.3 ± 2.6
Male sex — no. (%)	20 (80.0)	17 (68.0)	20 (74.1)
Right handedness — no. (%)	21 (84.0)	23 (92.0)	23 (85.2)
WAIS-R full scale IQ	136.8 ± 4.9	97.0 ± 9.8	98.6 ± 10.0

Mean and standard deviation values are denoted as mean ± standard deviation. SI, superior intelligence; AI, average intelligence; WAIS-R, Wechsler Adult Intelligence Scale-Revised; IQ, intelligence quotient.

In the current study, global network metrics including global efficiency, local efficiency, and network cost were calculated in each individual’s white matter structural network matrix and were compared between the groups. In addition, the network properties of rich club organization were also examined at an individual level. Network density and cost for the rich club, feeder, and local connection matrices were calculated and compared between the groups.

### Global Network Metrics

**Figure 1:**
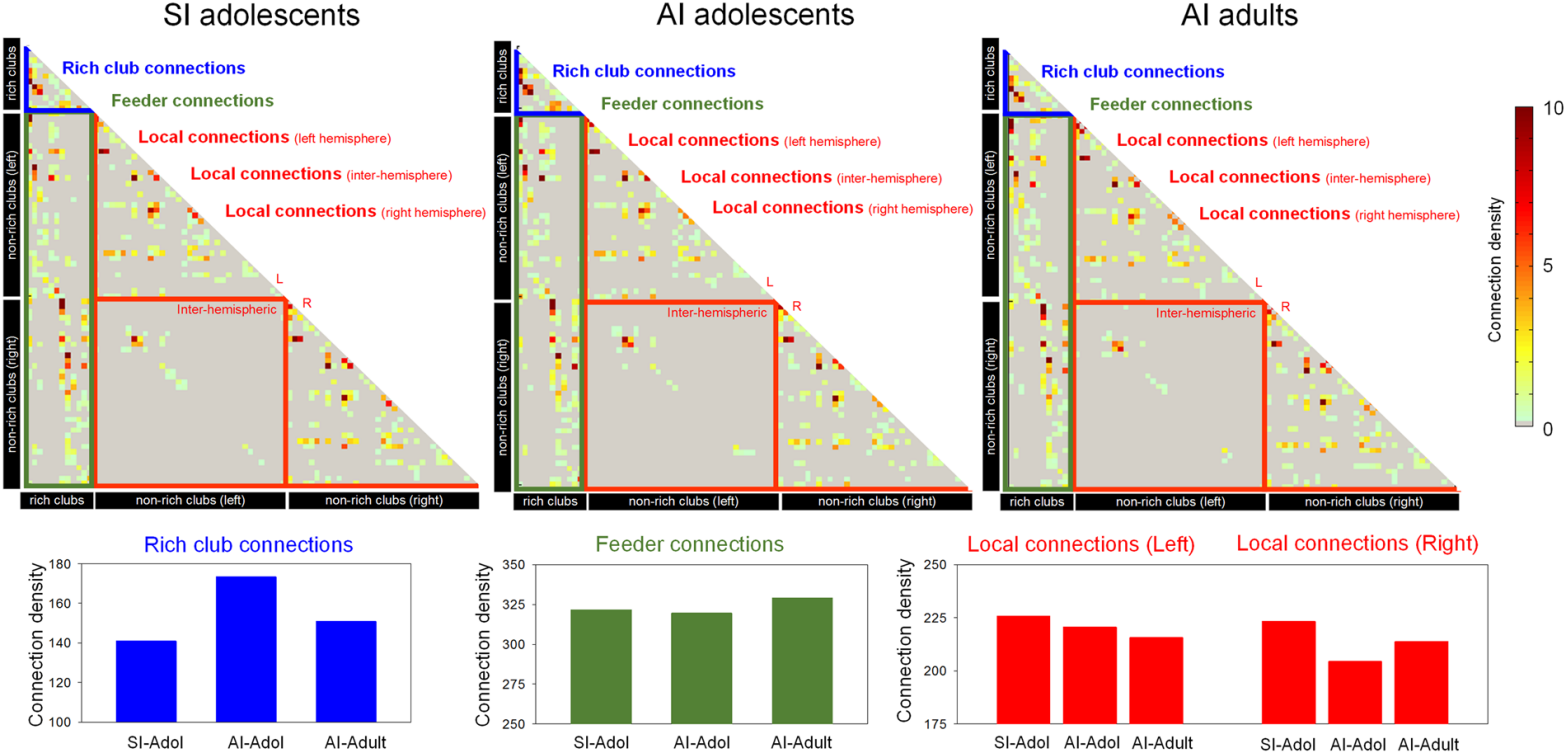
Group-averaged structural matrices of the SI-Adol (upper left), AI-Adol (upper middle), and AI-Adult (upper right) groups. Rows and columns of matrices (i.e., nodes) are ordered as the rich clubs, non-rich clubs of the left hemisphere, and non-rich clubs of the right hemisphere. For presentation purposes, the bar graphs, which represent the total connection density of rich club connection (blue), feeder connections (green), and local connections (red) of group-averaged matrix for each group, are provided in the lower panel.

We first assessed global network metrics including global efficiency, local efficiency, and network cost of the whole-brain structural connectivity matrix in each study group. The comparisons between the SI-Adol and AI-Adol groups may provide the information regarding the effects of *high intelligence* on network topology, while those between the AI-Adult and AI-Adol groups may provide information about the effects of age on network topology. Group-averaged structural matrix of each group is presented in Fig. 1.

**Figure 2:**
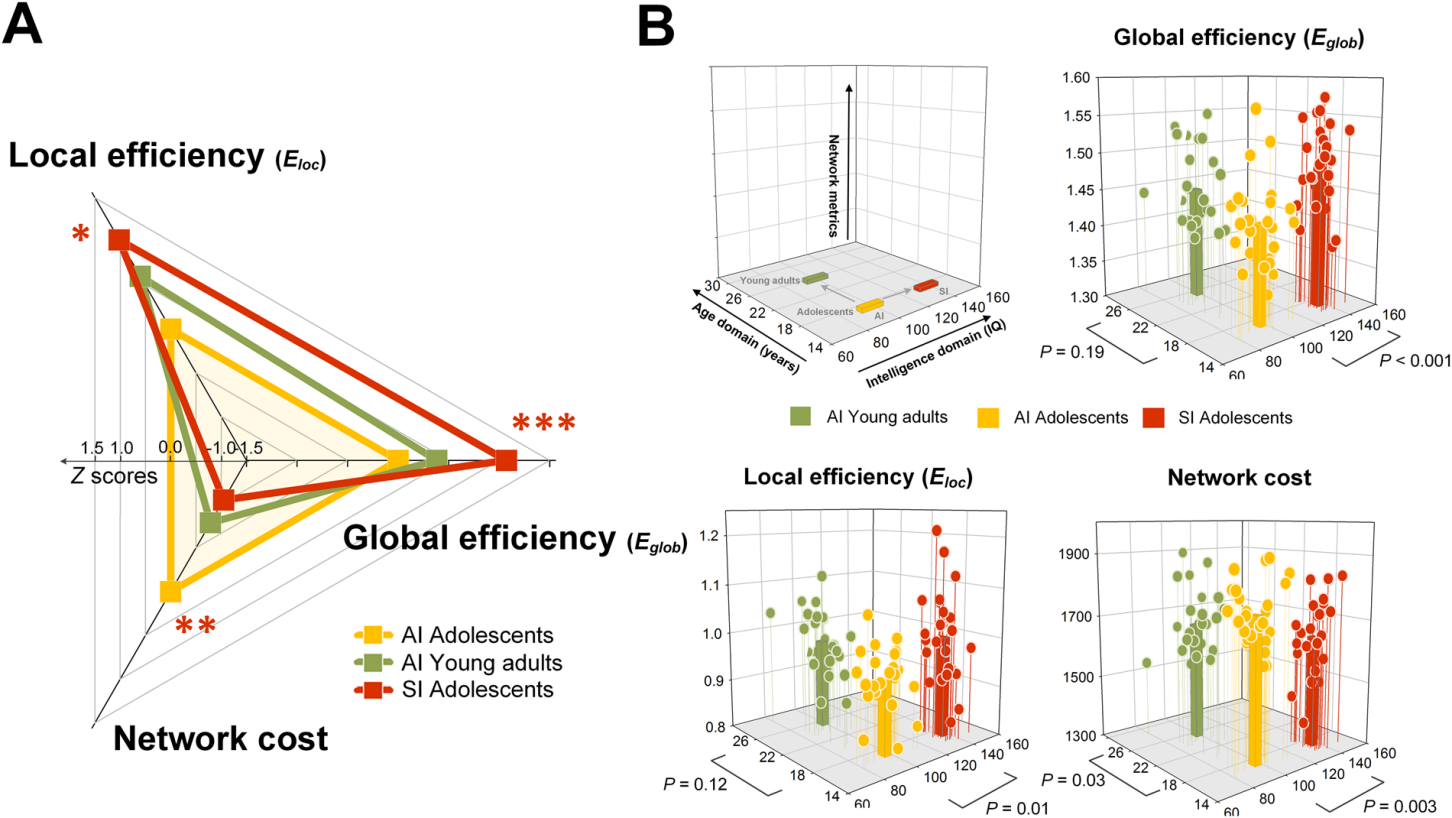
Global graph metrics of the study groups. (**A**) Standardized *Z* scores of global efficiency, local efficiency, and network cost were calculated using the means and standard deviations of the AI-Adol group and were plotted in the radar chart. Red asterisks indicate permutation-adjusted *P* values for the comparisons between the SI-Adol (red) and AI-Adol (yellow) groups, whereas green asterisks indicate those between the AI-Adult (green) and AI-Adol (yellow) groups. (**B**) Three-dimensional illustrations represent the individual plotting of global efficiency, local efficiency, and network cost in relations to age and intellectual quotient. The comparisons between the SI-Adol (red) and AI-Adol (yellow) groups may provide the information regarding the effects of high intelligence on network topology, while those between the AI-Adult (green) and AI-Adol (yellow) groups may represent the effects of age on network topology. *Permutation-adjusted *P* < 0.05; **permutation-adjusted *P* < 0.01; ***permutation-adjusted *P* < 0.001, 10,000 permutations.

The SI-Adol group had a higher level of global efficiency (*β* = 0.48, permutation-adjusted *P* = 0.0001) and local efficiency (*β* = 0.33, permutation-adjusted *P* = 0.01) than the AI-Adol group, implying greater efficiency of segregated and integrated information processing in adolescents with high intelligence (Fig. 2). In addition, a lower wiring cost was observed in the SI-Adol group, as compared with the AI-Adol group (*β* = −0.41, permutation-adjusted *P* = 0.003, Fig. 2), which suggests greater cost-effectiveness of information processing in the highly intelligent brain.

Results from comparing global network metrics between the AI-Adult and AI-Adol groups may reflect the age effects on global network metrics, such as, normative developmental changes in network measures. There were no significant differences in global efficiency (*β* = 0.21, permutation-adjusted *P* = 0.19) and local efficiency (*β* = 0.26, permutation-adjusted *P* = 0.12) of the whole-brain structural connectivity matrix between the AI-Adult and AI-Adol groups (Fig. 2). However, the AI-Adult group had a lower wiring cost than the AI-Adol group (*β* = −0.33, permutation-adjusted *P* = 0.03, Fig. 2), suggesting that the adolescent brain may become more cost-effective as it grows into adulthood.

### Network Metrics of Rich Club Organization

**Figure 3:**
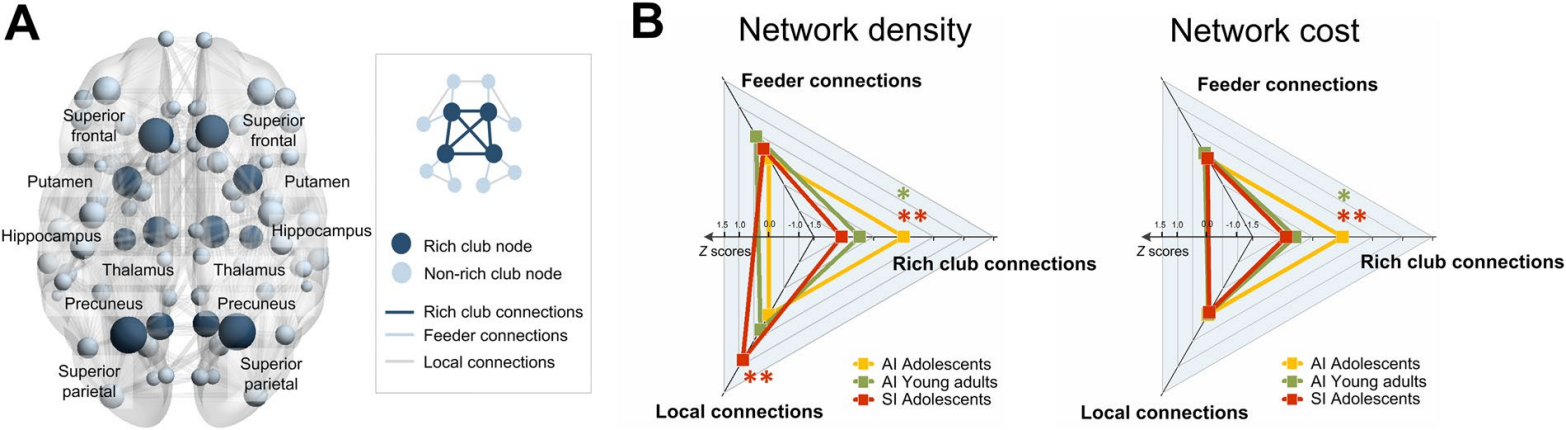
(**A**) Group-averaged reconstructed structural brain network represents rich club members including the bilateral superior frontal, superior parietal, precuneus, hippocampus, putamen, and thalamus (dark blue circles) and non-rich club members (light blue circles). Three-dimensional rendering of the brain network in the MNI space was generated using BrainNet viewer^49^. (**B**) Standardized *Z* scores of network density and cost for rich club, feeder, and local connections were calculated using the means and standard deviations of the AI-Adol group and were plotted in the radar chart. Red asterisks indicate permutation-adjusted *P* values for the comparisons between the SI-Adol (red) and AI-Adol (yellow) and groups, whereas green asterisks indicate those between the AI-Adult (green) and AI-Adol (yellow) groups. *permutation-adjusted *P* < 0.05; **permutation-adjusted *P* < 0.01, 10,000 permutations).

We found that the SI-Adol group exhibited reduced levels of network density (*β* = −0.44, permutation-adjusted *P* = 0.003, Fig. 3) of the rich club connection matrix, as compared with the AI-Adol group. Network cost of the rich club connection matrix was lower in the SI-Adol group than the AI-Adol group (*β* = −0.43, permutation-adjusted *P* = 0.004, Fig. 3). However, the local connection matrix of the SI-Adol group had a higher density level (*β* = 0.41, permutation-adjusted *P* = 0.006, Fig. 3) than that of the AI-Adol group. There were no differences in network density (*β* = 0.08, permutation-adjusted *P* = 0.61, Fig. 3) of the feeder connection matrix between the SI-Adol and AI-Adol groups. The levels of wiring cost of the feeder connection matrix (*β* = −0.02, permutation-adjusted *P* = 0.89, Fig. 3) and local connection matrix (*β* = −0.01, permutation-adjusted *P* = 0.93, Fig. 3) were similar between the SI-Adol and AI-Adol groups.

For the comparisons between the AI-Adult and AI-Adol groups, network density (*β* = −0.28, permutation-adjusted *P* = 0.03, Fig. 3) and cost (*β* = −0.31, permutation-adjusted *P* = 0.02, Fig. 3) of the rich club connection matrix were also lower in the AI-Adult group, as compared with the AI-Adol group. There were no differences in network metrics of the feeder connection matrix (network density, *β* = 0.17, permutation-adjusted *P* = 0.18; network cost, *β* = 0.04, permutation-adjusted *P* = 0.79) and local connection matrix (network density, *β* = 0.13, permutation-adjusted *P* = 0.37; network cost, *β* = −0.0005, permutation-adjusted *P* = 1.00) between the AI-Adult and AI-Adol groups (Fig. 3).

**Figure 4:**
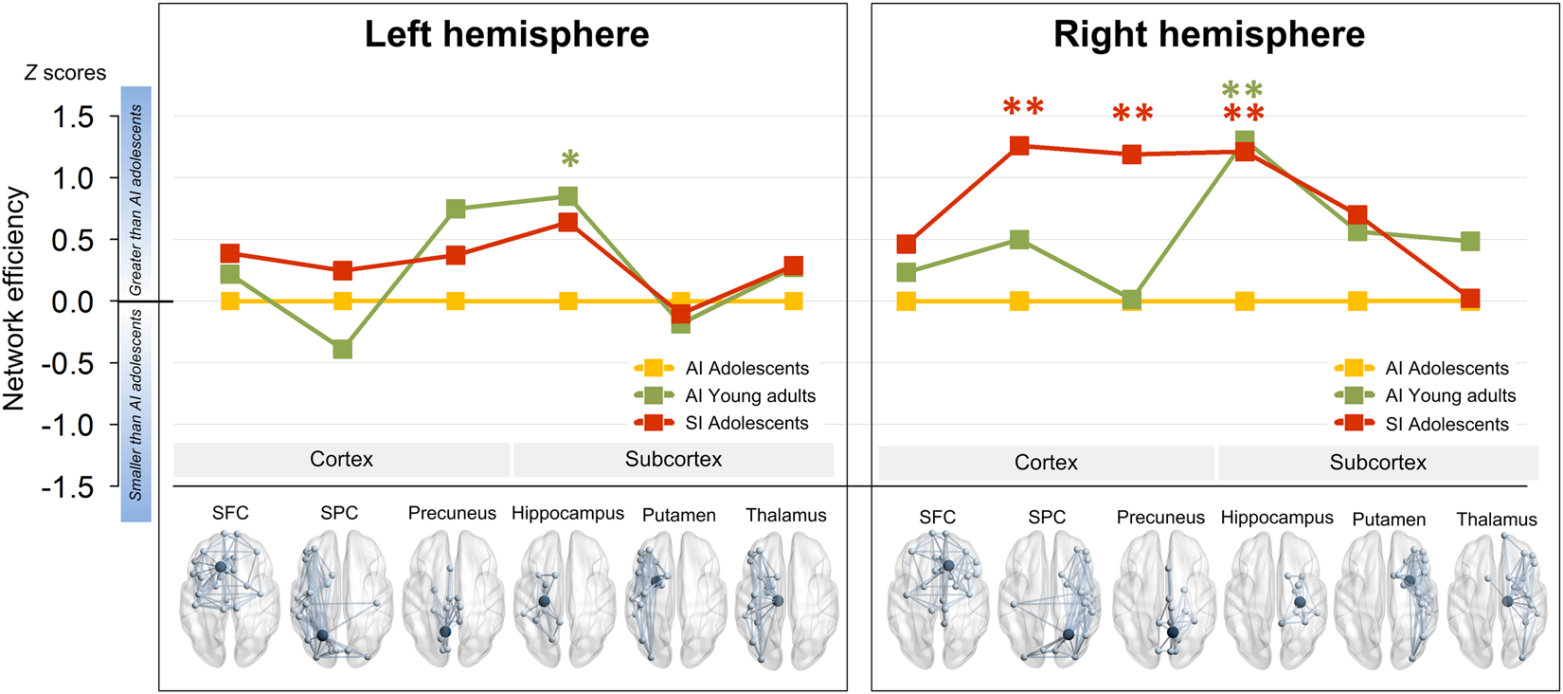
Standardized *Z* scores of network efficiency for feeder connections calculated using the means and standard deviations of the AI-Adol group. Red asterisks indicate permutation-adjusted *P* values for the comparisons between the SI-Adol (red) and AI-Adol (yellow) groups, whereas green asterisks indicate those between the AI-Adult (green) and AI-Adol (yellow) groups. *FDR-corrected *P* < 0.05; **FDR-corrected *P* < 0.01.

To further determine the segregating nature of connection network in each rich club nodes, network efficiency of each rich club’s feeder connection matrix was computed in the superior frontal cortex (SFC), superior parietal cortex (SPC), precuneus, hippocampus, putamen, and thalamus of each hemisphere (Fig. 4). Among 12 rich club nodes, network efficiency for feeder connections of the right parietal regions including the SPC (*β* = 0.52, false discovery rate (FDR)-corrected *P* = 0.009) and precuneus (*β* = 0.48, FDR-corrected *P* = 0.004) was greater in the SI-Adol group than in the AI-Adol group (Fig. 4). The feeder connection matrix of the right hippocampus was found to be more efficient in both SI-Adol (*β* = 0.48, FDR-corrected *P* = 0.004) and AI-Adult (*β* = 0.51, FDR-corrected *P* = 0.001) groups, as compared with the AI-Adol group (Fig. 4). Network efficiency of the left hippocampal feeder connection matrix was greater in the AI-Adult group (*β* = 0.38, FDR-corrected *P* = 0.02) but not in the SI-Adol group (*β* = 0.29, FDR-corrected *P* = 0.14) as compared with the AI-Adol group. There were no significant differences in efficiency for the feeder connection matrix of other rich club nodes between the groups.

To ensure the robustness of results, we repeated all analyses with connectivity matrices, which were constructed using different FA thresholds for each individual. We found that the overall results were similar irrespective of the FA thresholds (Supplementary Table 1). In addition, we investigated the group-differences in topological properties across a range of thresholds between 1% and 20% sparsity in 1% increments. We found that the results were not significantly influenced by the thresholding procedures (Supplementary Result 1 and Supplementary Figure 1).

Given the unequal sample size (25 AI-Adol vs. 27 AI-Adult), the robustness of the results was examined using the jackknife resampling procedure^26^. Each individual was excluded from the analysis, one at a time accordingly. The results from this resampling method for the group-differences in network metrics between the AI-Adol and the AI-Adult groups did not change (Supplementary Result 2).

For comparison purposes, the group-differences in network metrics of rich club organization were examined with the information of the rich club and non-rich club members, which were selected based on previous studies. We repeated the analyses using the information from the rich club members, which were defined as the top 12 highest ranking nodes based on the degrees of the group-averaged structural networks of each AI-Adol, SI-Adol, and AI-Adult group. The results from these repeated analyses were similar to the main analyses, showing that the main results are not influenced by the group-specific location of the rich club members (Supplementary Table 2).

Furthermore, we repeated multiple regression analyses including a measure of intelligence (IQ) as an independent variable in an auxiliary analysis to examine the effects of high intelligence. Sex composition was included as a relevant covariate. The results remained similar for all repeated analyses (Supplementary Result 3).

## Discussion

Recently, architectural features of the whole-brain structural connectivity network have been proposed to be associated with intelligence^4^, as opposed to previous reports which have focused on the anatomical characteristics of particular brain regions^8^. Although several studies have assessed the relationships between general intelligence and network metrics in the general population^9–12^, this is the first study to characterize the neural correlates of intellectual giftedness from a network perspective in the developing brain. In the current study, we found that high level integration of the whole-brain structural connectivity network was associated with high intelligence. Interestingly, even with higher global efficiency, economic network topology such as a lower wiring cost was found in intellectually gifted adolescents relative to normal adolescents. These findings support previous observations which suggested that high levels of global information integration between brain regions may account for superior performance on a test of intelligence for those with normally distributed intelligence scores^9–12^.

Although the previous findings did not show a clear relationship between local clustering of connections and IQ in the general population^10–12^, we found that adolescents with high intelligence possessed the network topology of efficient locally segregated processing. Intellectually gifted adolescents, relative to normal adolescents with average intelligence, showed a higher level of local efficiency, implying a highly segregated network architecture. Highly efficient whole-brain structural connectivity network with highly clustering structures, which was observed in the gifted adolescents, may confer an advantage in converging the diverse information derived from each brain area both accurately and quickly. A recent study defined general intelligence as a fractal output by leveraging multiple anatomically distinct systems, rather than as one general function derived from all regions across the entire brain^27^. In this regard, the role of highly specialized and segregated network topology may be more important than previously thought in explaining the highly intelligent brain.

Converging lines of evidence have indicated that integration of information from the distributed brain regions may require two aspects of network organization: one based on global efficiency of communication among specialized brain regions and the other based on a specific set of brain regions such as hubs and their respective connections^28, 29^. In particular, the ‘richness’ of hub nodes in structural connectivity network may contribute to efficient integration of dispersed information^29^. Therefore, it may be expected that intellectual ability is associated not only with global efficiency but also with rich club connections^11^. However, we found that the efficiency and cost of rich club connections were lower in highly intelligent adolescents than in adolescents with average intelligence. Rather, the network efficiency of local connection matrix interconnecting non-rich club regions was higher in highly intelligent adolescents than in adolescents with average intelligence. These findings may be explained by the specific network characteristics of information processing between brain regions in the highly intelligent brain, which may be unique and potentially different from those in the normal brain.

In order to deal with any potential or unpredictable dangers and opportunities efficiently, the network dynamics of the human brain may quickly and automatically adapt to resolve ambiguities^30^. For instance, in the face of a salient stimuli, the brain may disrupt or break existing topological properties and link to other segregated communities specific for addressing the demanding stimuli^30, 31^. Rich club connections, as core connections, may constitute a potential anatomical substrate for this dynamic cooperation process of the brain^29^. The human brain may evolve to have such a dynamic network architecture, in order to cope with and predict for the unknowns^30, 31^. However, given that creative cognitive processes may occur spontaneously, and not triggered by any conscious volition^32^, the highly intelligent and creative brain may not entirely follow this efficient, yet somewhat stereotypical, process of information integration that may be mediated by rich club connections. Furthermore, highly intelligent adolescents appear to utilize or obtain more information from multiple locally segregated connections. This finding may be supported by a recent study underscoring that the efficiency of weak brain connections, rather than that of strong brain connections, is responsible for general cognitive function^33^. In addition, recent reviews indicated that creative thinking may depend more on randomly diffused brain involvement rather than localized and sequential processes^32, 34^. In summary, a highly intelligent brain may have a higher level of efficiency in integrating information among brain regions, but be less dependent on rich club connections for global efficiency.

As a final point, we found that highly intelligent adolescents may have a more efficient subnetwork centered on the right parietal cortical regions including the SPC and precuneus than normal adolescents. Non-rich club regions that were structurally connected with the SPC were primarily located in the fronto-parietal area, while the precuneus was predominantly connected with non-rich club nodes including the medial frontal and temporal regions (Supplementary Table 3). These feeder connection networks of the SPC and precuneus may closely resemble the resting-state frontoparietal and default mode networks, respectively^35^. A large-scale functional neuroimaging study has also recently reported that connections among the brain regions in the frontoparietal and default mode networks were associated with measures of general intelligence^36^. Taken together^23, 36, 37^, the current findings imply that efficient frontoparietal and default mode network topology play an important role in intelligence and creativity.

Although the level of global efficiency of the whole-brain structural connectivity network was similar between the groups, young adults with AI demonstrated a more cost-effective network topology in comparison with adolescents with AI. This finding may be tentatively interpreted that the brain develops in a manner which minimizes the physical cost of wiring a complex network throughout late adolescence to young adulthood. Given this finding, the characteristic network topology to enhance both segregated and integrated information processing as observed in intellectually gifted adolescents may not merely reflect developmental promotion.

Several limitations should be considered when interpreting the results. Although young adults with average intelligence were included as a comparison group for determining potential effects of age on network topology, the current cross-sectional design could not characterize the developmental network trajectory of highly intelligent adolescent that may be different from that of normal adolescents. Furthermore, although IQ has been regarded as a widely used measure for general intelligence^7^, it should be noted that there are other measures that assesses different aspects and levels of one’s intelligence. Therefore, the current results should be replicated in future studies with the use of additional diverse measures for intelligence. Considering region-specificity and non-linearity of developmental trajectory in the structural connectivity network^38, 39^, future longitudinal research is needed to follow up with network topology for intellectually gifted adolescents versus non-gifted adolescents. This will then provide evidence for a unique trajectory across the developmental lifespan in those who are intellectually gifted. Similar to other studies examining structural connectivity network using deterministic tractography, it should be noted that there are various methodological options to define node, edge, and network construction. The issue of crossing fiber may also be present in using deterministic tractography^40^. Our findings suggest that young adults showed less efficient but more cost-effective rich club connections relative to normal adolescents. Although the histological background could not be determined in this study, this finding may be attributed, in part, to the developmental process of pruning of the white matter connections and decrease in the fiber density. It should be noted that the current results were derived from the cross-sectional group comparisons. Therefore, future longitudinal studies that examine the effects of aging and IQ on network metrics at a within-subject level would be necessary.

In the current study, we characterized the specific network attributes underlying high intelligence by investigating adolescents with an IQ score of greater than 130 and adolescent with an IQ score of less than 120. Our findings suggest that the intelligent brain may not only be strongly integrated but also highly segregated in the context of network topology. Furthermore, the brain regions of highly intelligent adolescents, relative to those of normal adolescents, may communicate more extensively, while being less dependent on rich club communications.

## Materials and Methods

### Participants and Intelligence Assessments

Adolescents who attended high schools for intellectually gifted students in South Korea and were referred by their teachers for their intellectual giftedness, based on their academic performance, were included as the SI-Adol group. Given that IQ is considered a reliable and valid measure for intelligence^7^, the participants’ intelligence was assessed using the Wechsler Adult Intelligence Scale-Revised (WAIS-R). In general, IQ, as measured using the WAIS-R, is classified into the following groups: very superior (130 and above), superior (120–129), average (80–119), borderline (70–79), and very low (69 and below)^41^. In accordance with this criteria, adolescents with the general intelligence score of IQ 130 and higher were categorized into the SI-Adol group (n = 25) and adolescents with IQ < 120 were categorized into the AI-Adol group (n = 25). All adolescent participants were between 15 and 19 years old. Twenty-seven young adults with an IQ < 120 were included as the AI-Adult group. Study participants with a current or past history of major medical, neurological, or psychiatric illness were excluded from the study. History of head injury with loss of consciousness or contraindications to magnetic resonance imaging (MRI) were also the exclusion criteria.

This study was approved by the Institutional Review Board of the Catholic University of Korea College of Medicine, and all procedures were performed in accordance with institutional and national guidelines and regulations. All participants and their legal guardians provided written informed consent/assent after receiving a complete description of the study.

### Image Acquisition and Processing

Study participants underwent T1-weighted and diffusion-weighted MRI scanning using a 1.5-Tesla whole-body imaging system (Signa HDx, GE Healthcare, Milwaukee, WI). High-resolution T1-weighted structural images were obtained using a three-dimensional spoiled gradient echo sequence with the following acquisition parameters: 256 × 256 image matrix, repetition time = 24 ms, echo time = 5 ms, field of view (FOV) = 240 mm, flip angle = 45°, number of excitation (NEX) = 2, slice thickness = 1.2 mm, no skip. Diffusion-weighted images with 54 non-colinear directions (b = 1000 s/m^2^) were acquired with the acquisition parameters of 96 × 96 image matrix, repetition time = 17,000 ms, echo time = 84 ms, FOV = 220 mm, flip angle = 90°, NEX = 2, slice thickness = 2.3 mm, no skip. In addition, six images without diffusion weighting (b = 0 s/m^2^) were acquired. For screening purposes, axial fluid-attenuated inversion recovery images (256 × 192 image matrix, repetition time = 8,802 ms, echo time = 88 ms, inversion time = 2,200 ms, FOV = 220 mm, flip angle = 90°, NEX = 1, slice thickness = 5 mm, no skip) and T2-weighted images (256 × 192 image matrix, repetition time = 2,817 ms, echo time = 26 ms, FOV = 220 mm, flip angle = 90°, NEX = 1, slice thickness = 5 mm, no skip) were also acquired.

Preprocessing of T1-weighted images was performed to be used as the anatomical reference and for selecting nodes of structural brain network. Each individual T1-weighted image was parcellated into 34 cortical and 7 subcortical regions per each hemisphere using the FreeSurfer tool (http://surfer.nmr.mgh.harvard.edu)^42^. A set of 82 cortical and subcortical regions-of-interests (ROIs) was selected for representing the nodes of structural brain network^15^. A rater (J.M.) who was blind to the group assignment performed a visual inspection for all images to validate the cortical and subcortical segmentation and anatomical labels as well as correct misclassification manually. An averaged non-diffusion image (b = 0 s/m^2^) of each individual was co-registered to the respective T1-weighted image using affine transformation. All cortical and subcortical ROIs in the native space were inversely transformed to the diffusion space.

Preprocessing of diffusion-weighted images included the realignment with the averaged non-diffusion image (b = 0 s/m^2^) and the correction for head motion and eddy current distortions. Diffusion tensor was fitted and fractional anisotropy (FA) values were computed within each voxel using the Diffusion Toolkit (http://trackvis.org/).

### Network Construction

Detailed information on network construction is described elsewhere^17^. In brief, deterministic fiber tracking based on the Fiber Assignment by Continuous Tracking (FACT) algorithm was applied to reconstruct white matter tract^43^ and fiber connectivity among the 82 ROIs was then calculated. Three seeds in each voxel within the ROIs were started and each streamline from the seed followed the principal diffusion direction from one voxel to the next. A streamline was terminated with the criteria of reaching a voxel with a FA value < 0.1, having a turning angle of >45 degrees, or exceeding the ROI. Through these processes implemented with the Trackvis (http://trackvis.org) software package, all fiber tracts interconnecting ROIs were reconstructed. In order to exclude obvious spurious connections, we considered all streamlines longer than 10 mm as being anatomically connected.

Fiber tracts interconnecting 82 cortical and subcortical nodes were combined to reconstruct the structural brain network comprising a set of nodes (reflecting cortical and subcortical ROIs) and edges (reflecting reconstructed connections between nodes). A minimum of 3 streamlines interconnecting two different nodes were required to be considered as being structurally connected in order to eliminate potentially spurious connections and thereby to construct network matrix in order to eliminate potentially spurious connections. Network edges were weighted according to streamline density, which was computed as the number of reconstructed streamlines between two nodes. The number of reconstructed streamlines was divided by total streamline counts to yield the normalized streamline density. Weighted connectivity network of each subject constructed based on normalized streamline density was finally used to assess its graph metrics.

### Assessment of Global Network Metrics

The Brain Connectivity Toolbox (http://www.brain-connectivity-toolbox.net) was used to perform network analysis. For the assessment of graph metrics of the global topological organization of each whole-brain structural connectivity network, global efficiency (*E*_*glob*_) and local efficiency (*E*_*loc*_) were computed in each matrix. Global efficiency is a measure of integration of structural connectivity network and defined as the average inverse shortest length between all pairs of nodes, whereas local efficiency is a measure of segregation of structural connectivity network and defined as the efficiency computed on node neighborhoods^44, 45^. Network cost was calculated as the streamline density multiplied by the average length of the reconstructed streamlines^46^.

### Assessment of Rich Club Organization

Based on previous studies^15, 47^, rich club nodes were selected as follows: the SFC, SPC, precuneus, hippocampus, putamen, and thalamus, all bilaterally. Rich club nodes are more likely to be connected with each other than expected by chance^15^. All connections between nodes in each structural matrix were categorized into one of following: ‘rich club connections’ which are defined as the sum of all connections linking rich club nodes, ‘feeder connections’ which are defined as the sum of connections linking rich club to non-rich club nodes, and ‘local connections’ which are defined as the sum of connections linking non-rich club nodes^15^. We examined network density for the rich club, feeder, and local connection matrix by calculating the sum of streamline density of each connection matrix. Network cost for each rich club, feeder, and local connection matrix was also computed.

### Statistical Analysis

Group effects on global network metrics including global efficiency, local efficiency, and network cost of the whole-brain structural connectivity network were examined in each “SI-Adol group vs. AI-Adol group” and “AI-Adult group vs. AI-Adol group” using multiple linear regression analysis, while adjusting for sex. The first set of comparison may provide information regarding the effects of high intelligence on network topology, while the effects of age on network topology could be examined in the second set of comparison. In addition, multiple linear regression analysis with sex as a covariate was performed on both “SI-Adol group vs. AI-Adol group” and “AI-Adult group vs. AI-Adol group” to examine the group effects on network metrics of rich club organization including network density and network cost for each rich club, feeder, and local connection matrix. We calculated the permutation-adjusted *P* values for each network measure^48^. A total of 10,000 permutations were performed to obtain an empirical null distribution of effects under the null-hypothesis. A permutation-adjusted *P* value was computed based on the proportion of permutations with *P* values under the null distribution that was greater than the observed values from the actual data set^48^.

Lastly, the segregation nature of the connection network of each rich club member was explored. Non-rich club regions connected with each rich club were selected on the basis of group-averaged reconstructed network (Supplementary Table 3). The group-averaged network was reconstructed using a threshold which only includes connections found in at least 50% of the subjects and by averaging the cell values of individual matrices. Using the multiple linear regression analysis, group effects on network efficiency of feeder connection network were examined in each rich club member - the bilateral SFC, SPC, precuneus, hippocampus, putamen, and thalamus - after controlling for sex. Results for the feeder connection matrix of each rich club node were FDR-corrected and findings surviving the FDR-correction were considered as being statistically significant.

## Electronic supplementary material

Supplementary Information

## References

[1] Gottfredson LS (1997). Mainstream science on intelligence: an editorial with 52 signatories, history, and bibliography. Intelligence.

[2] Deary IJ (2012). Intelligence. Annu. Rev. Psychol..

[3] McDaniel M (2005). Big-brained people are smarter: A meta-analysis of the relationship between *in vivo* brain volume and intelligence. Intelligence.

[4] Colom R, Karama S, Jung RE, Haier RJ (2010). Human intelligence and brain networks. Dialogues Clin. Neurosci..

[5] Deary IJ, Penke L, Johnson W (2010). The neuroscience of human intelligence differences. Nat. Rev. Neurosci..

[6] Gray JR, Thompson PM (2004). Neurobiology of intelligence: science and ethics. Nat. Rev. Neurosci..

[7] Shaw P (2007). Intelligence and the developing human brain. Bioessays.

[8] Jung RE, Haier RJ (2007). The Parieto-Frontal Integration Theory (P-FIT) of intelligence: converging neuroimaging evidence. Behav. Brain. Sci..

[9] Li Y (2009). Brain anatomical network and intelligence. PLoS Comput. Biol..

[10] Fischer FU, Wolf D, Scheurich A, Fellgiebel A (2014). Association of structural global brain network properties with intelligence in normal aging. PLoS One.

[11] Kim DJ (2016). Children’s intellectual ability is associated with structural network integrity. Neuroimage.

[12] van den Heuvel MP, Stam CJ, Kahn RS, Hulshoff Pol HE (2009). Efficiency of functional brain networks and intellectual performance. J. Neurosci..

[13] Song M (2008). Brain spontaneous functional connectivity and intelligence. Neuroimage.

[14] Cole MW, Yarkoni T, Repovs G, Anticevic A, Braver TS (2012). Global connectivity of prefrontal cortex predicts cognitive control and intelligence. J. Neurosci..

[15] van den Heuvel MP, Sporns O (2011). Rich-club organization of the human connectome. J. Neurosci..

[16] Bullmore E, Sporns O (2012). The economy of brain network organization. Nat. Rev. Neurosci..

[17] Yoon S (2016). Effects of creatine monohydrate augmentation on brain metabolic and network outcome measures in women with major depressive disorder. Biol. Psychiatry.

[18] Mrazik M, Dombrowski SC (2010). The Neurobiological foundations of giftedness. Roeper Review.

[19] Diamond MC, Scheibel AB, Murphy GM, Harvey T (1985). On the brain of a scientist: Albert Einstein. Exp. Neurol..

[20] Witelson SF, Kigar DL, Harvey T (1999). The exceptional brain of Albert Einstein. Lancet.

[21] Falk D, Lepore FE, Noe A (2013). The cerebral cortex of Albert Einstein: a description and preliminary analysis of unpublished photographs. Brain.

[22] Men W (2014). The corpus callosum of Albert Einstein’s brain: another clue to his high intelligence?. Brain.

[23] Lee KH (2006). Neural correlates of superior intelligence: stronger recruitment of posterior parietal cortex. Neuroimage.

[24] Desco M (2011). Mathematically gifted adolescents use more extensive and more bilateral areas of the fronto-parietal network than controls during executive functioning and fluid reasoning tasks. Neuroimage.

[25] Newman, T. M. “Assessment of giftedness in school-age children using measures of intelligence or cognitive abilities”, in *Handbook of* Giftedness *in Children* (ed. Pfeiffer, S. I.) 161–176 (Springer, 2008).

[26] Efron, B. *The Jackknife*, *the Bootstrap and Other Resampling Plans* (Society for Industrial and Applied Mathematics, 1982).

[27] Hampshire A, Highfield RR, Parkin BL, Owen AM (2012). Fractionating human intelligence. Neuron.

[28] Sporns O (2013). Network attributes for segregation and integration in the human brain. Curr. Opin. Neurobiol..

[29] van den Heuvel MP, Sporns O (2013). Network hubs in the human brain. Trends Cogn. Sci..

[30] Dehaene S, Changeux JP (2011). Experimental and theoretical approaches to conscious processing. Neuron.

[31] Baars BJ (2002). The conscious access hypothesis: origins and recent evidence. Trends. Cogn. Sci..

[32] Wiggins GA, Bhattacharya J (2014). Mind the gap: an attempt to bridge computational and neuroscientific approaches to study creativity. Front. Hum. Neurosci..

[33] Santarnecchi E, Galli G, Polizzotto NR, Rossi A, Rossi S (2014). Efficiency of weak brain connections support general cognitive functioning. Hum. Brain. Mapp..

[34] Dietrich A, Kanso R (2010). A review of EEG, ERP, and neuroimaging studies of creativity and insight. Psychol. Bull..

[35] Smith SM (2009). Correspondence of the brain’s functional architecture during activation and rest. Proc. Natl. Acad. Sci. USA.

[36] Smith SM (2015). A positive-negative mode of population covariation links brain connectivity, demographic and behavior. Nature Neurosci..

[37] Jung RE, Mead BS, Carrasco J, Flores RA (2013). The structure of creative cognition in the human brain. Front. Hum. Neurosci..

[38] Wierenga LM (2016). The development of brain network architecture. Hum. Brain. Mapp..

[39] Baker ST (2015). Developmental changes in brain network hub connectivity in late adolescence. J. Neurosci..

[40] Mori S, van Zijl PC (2002). Fiber tracking: principles and strategies - a technical review. NMR Biomed..

[41] Weiss, L. G., Saklofske, D. H., Prifitera, A., Holdnack, J. A. *WISC-IV Advanced Clinical Interpretation* (Academic Press, 2006).

[42] Desikan RS (2006). An automated labeling system for subdividing the human cerebral cortex on MRI scans into gyral based regions of interest. Neuroimage.

[43] Mori S, Crain BJ, Chacko VP, van Zijl PC (1999). Three-dimensional tracking of axonal projections in the brain by magnetic resonance imaging. Ann. Neurol..

[44] Watts DJ, Strogatz SH (1998). Collective dynamics of ‘small-world’ networks. Nature.

[45] Rubinov M, Sporns O (2010). Complex network measures of brain connectivity: uses and interpretations. Neuroimage.

[46] van den Heuvel MP, Kahn RS, Goni J, Sporns O (2012). High-cost, high-capacity backbone for global brain communication. Proc. Natl. Acad. Sci. USA.

[47] Markett, S. *et al*. Serotonin and the brain’s rich club-association between molecular genetic variation on the TPH2 gene and the structural connectome. *Cereb*. *Cortex*. doi:10.1093/cercor/bhw059 (2016). 10.1093/cercor/bhw059 26975194

[48] Westfall, P. H. & Young, S. S. *Resampling-based Multiple Testing: Examples and Methods for P-value Adjustment* (John Wiley & Sons, 1993).

[49] Xia M, Wang J, He Y (2013). BrainNet Viewer: a network visualization tool for human brain connectomics. PLoS One.

