# Supplementary material for: Network attributes underlying intellectual giftedness in the developing brain
Source: Sci Rep. 2017 Sep 12;7:11321. doi: 10.1038/s41598-017-11593-3 (PMC5596014; doi:10.1038/s41598-017-11593-3)
Supplement: Supplementary file 1 — Supplementary Information [file 41598_2017_11593_MOESM1_ESM.pdf]

Supplementary Information

Network attributes underlying intellectual giftedness in the developing brain

Jiyoung Ma[1,2,+], Hee Jin Kang[1,3,+], Jung Yoon Kim[1,], Hyeonseok S. Jeong[4], Jooyeon Jamie Im[1,2], Eun Namgung[1,3], Myeong Ju Kim[1,3], Suji Lee[1,3], Tammy D. Kim[1], Jin Kyoung Oh[4], Yong-An Chung[4], In Kyoon Lyoo[1,3,5], Soo Mee Lim[6,*], and Sujung Yoon[1,3,*]

[1]Ewha Brain Institute, Ewha W. University, Seoul, South Korea
[2]Interdisciplinary Program in Neuroscience, College of Natural Sciences, Seoul National University, Seoul, South Korea
[3]Department of Brain and Cognitive Sciences, Ewha W. University, Seoul, South Korea,
[4]Department of Radiology, Incheon St. Mary's Hospital, College of Medicine, The Catholic University of Korea, Seoul, South Korea
[5]College of Pharmacy, Graduate School of Pharmaceutical Sciences, Ewha W. University, Seoul, South Korea
[6]Department of Radiology, Ewha W. University College of Medicine, Seoul, South Korea

[+]these authors contributed equally to this study.
[*]address for correspondence to Sujung Yoon and Soo Mee Lim, Ewha Brain Institute and Department of Brain and Cognitive Sciences, Ewha W. University, 52 Ewhayeodae-gil, Seodaemun-gu, Seoul 03760, South Korea. Email: sujungjyoon@ewha.ac.kr, Tel: +82-2-3277-2478 (S.Y.); Email: soomee@ewha.ac.kr, Tel: +82-2-2650-6022 (S.M.L.).

Supplementary Result 1

In this study, we evaluated the group-differences in topological properties across a range of threshold between 1% and 20% sparsity in 1% increments. This approach may minimize the effects of possible discrepancies in overall streamline density between the groups, which may originate from the developmental differences in pruning fiber tracts, and then enables us to explore differences in the intrinsic properties of the graphs[1,2,3]. Network metrics were calculated at each sparsity threshold. For all network metrics, we computed the areas under the curve (AUCs) across the range of sparsity thresholds. Multiple linear regression analysis with sex as a covariate was performed to examine between-group differences in the AUCs of network metrics (AI-Adol vs. SI-Adol groups; AI-Adol vs. AI-Adult groups). We found that the results were not significantly influenced by this thresholding procedure. There were significant group differences in global network metrics including global efficiency (β = 0.44, P = 0.002), local efficiency (β = 0.37, P = 0.008), and network cost (β = -0.44, P = 0.002) between the AI-Adol and SI-Adol groups. The AI-Adult group showed a lower wiring cost (β = -0.35, P = 0.006) as compared with the AI-Adol group. However, there were no differences in global efficiency (β = 0.18, P = 0.19) and local efficiency (β = 0.25, P = 0.07) between the AI-Adol and AI-Adult groups.

For network metrics of rich club organization, there were significant differences in network density and cost of the rich club connection matrix between the AI-Adol and SI-Adol groups (rich club connection density, β = -0.44, P = 0.001; rich club connection cost, β = -0.43, P = 0.002) as well as between the AI-Adol and AI-Adult groups (rich club connection density, β = -0.29, P = 0.03; rich club connection cost, β = -0.31, P = 0.02)(Supplementary Figure 1). Network density of the local connection matrix was higher in the SI-Adol group as compared with the AI-Adol group (β = 0.35, P = 0.01), while this difference was not found between the AI-Adol and AI-adult groups (β = 0.07, P = 0.60)(Supplementary Figure 1). There were no differences in network cost of the local connection matrix between the AI-Adol and SI-Adol groups (β = -0.05, P = 0.75) as well as between the AI-Adol and AI-Adult groups (β = -0.03, P = 0.81). The levels of density

and cost of the feeder connection matrix were similar between the AI-Adol and SI-Adol groups (feeder connection density, β = 0.05, P = 0.74; feeder connection cost, β = -0.03, P = 0.83) as well as between the AI-Adol and AI-Adult groups (feeder connection density, β = 0.15, P = 0.27; feeder connection cost, β = 0.03, P = 0.84)(Supplementary Figure 1).

Supplementary Result 2

Using the jackknife resampling procedure, we repeated analyses for the comparison between the AI-Adult and AI-Adol groups to exclude each subject one at a time. The results remained unchanged in all cases, where there were no significant differences in global efficiency (regression coefficient ± standard error [SE] = 0.022 ± 0.015, P = 0.15) and local efficiency (regression coefficient ± SE = 0.031 ± 0.016, P = 0.06) of the whole-brain structural connectivity matrix between the AI-Adult and AI-Adol groups. In contrast, the AI-Adult group showed a lower wiring cost relative to the AI-Adol group (regression coefficient ± SE = -66.4 ± 26.1, P = 0.01). In regard to the network metrics of rich club organization, the network density (regression coefficient ± SE = -22.0 ± 9.9, P = 0.03) and cost (regression coefficient ± SE = -2921.5 ± 1199.2, P = 0.02) of the rich club connection matrix were lower in the AI-Adult group than in the AI-Adol group. There were no differences in network metrics of feeder (network density, regression coefficient ± SE = 13.4 ± 10.3, P = 0.20; network cost, regression coefficient ± SE = 204.0 ± 785.0, P = 0.80) and local (network density, regression coefficient ± SE = 8.61 ± 9.37, P = 0.36; network cost, regression coefficient ± SE = -3.37± 893.2, P = 1.00) connection matrices between the AI-Adol and AI-Adult groups.

Supplementary Result 3

Auxiliary analyses using a measure of IQ as an independent variable among adolescent participants demonstrated statistically significant effects of global efficiency (β = 0.42, P = 0.002), local efficiency (β = 0.29, P = 0.04), and network cost (β = -0.36, P = 0.01) on IQ, respectively. In addition, network density (β = -0.46, P = 0.001) and cost (β = -0.44,

P = 0.002) of the rich club connection matrix significantly predicted for IQ, respectively, among adolescent participants.

Supplementary Table 1. Group difference in network metrics with different fractional anisotropy (FA) thresholds

**Network metrics**	***β* values (*P* values)**		
	FA threshold = 0.1	FA threshold = 0.15	FA threshold = 0.2
AI-Adol group vs. SI-Adol group			
Global efficiency	0.48 (0.001)	0.48 (0.001)	0.28 (0.05)
Local efficiency	0.33 (0.02)	0.23 (0.12)	0.05 (0.72)
Network cost	-0.41 (0.004)	-0.26 (0.08)	-0.21 (0.15)
Rich club connections density	-0.44 (0.001)	-0.40 (0.003)	-0.34 (0.02)
Rich club connections cost	-0.42 (0.002)	-0.35 (0.01)	-0.29 (0.04)
Feeder connections density	0.08 (0.58)	0.11 (0.45)	0.10 (0.51)
Feeder connections cost	-0.02 (0.89)	0.09 (0.54)	0.14 (0.35)
Local connections density	0.41 (0.004)	0.33 (0.02)	0.30 (0.04)
Local connections cost	-0.01 (0.93)	0.11 (0.45)	0.10 (0.47)
AI-Adol group vs. AI-Adult group			
Global efficiency	0.21 (0.13)	0.30 (0.03)	0.10 (0.50)
Local efficiency	0.26 (0.06)	0.21 (0.13)	0.02 (0.86)
Network cost	-0.33 (0.01)	-0.27 (0.047)	-0.19 (0.14)
Rich club connections density	-0.28 (0.03)	-0.27 (0.03)	-0.29 (0.03)
Rich club connections cost	-0.31 (0.02)	-0.29 (0.02)	-0.28 (0.03)
Feeder connections density	0.17 (0.20)	0.23 (0.08)	0.20 (0.12)
Feeder connections cost	0.04 (0.79)	0.14 (0.30)	0.22 (0.11)
Local connections density	0.13 (0.36)	0.04 (0.78)	0.14 (0.33)
Local connections cost	-0.00 (1.00)	-0.03 (0.86)	0.04 (0.76)

Supplementary Table 2. Group difference in network metrics with different rich club and non-rich club members from each group

Group-averaged structural brain network of each group	Network metrics	β values (*P* values)	
		AI-Adol vs. SI-Adol	AI-Adol vs. AI-Adult
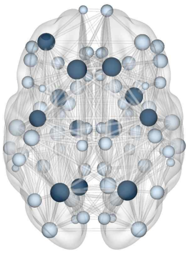	Rich club connections density	-0.38 (0.007)	-0.29 (0.03)
	Rich club connections cost	-0.35 (0.01)	-0.37 (0.006)
	Feeder connections density	0.04 (0.78)	-0.04 (0.77)
	Feeder connections cost	-0.11 (0.46)	-0.15 (0.30)
	Local connections density	0.38 (0.007)	0.33 (0.01)
[AI-Adol group]	Local connections cost	-0.06 (0.67)	0.15 (0.31)
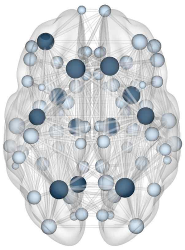	Rich club connections density	-0.43 (0.002)	-0.22 (0.10)
	Rich club connections cost	-0.38 (0.008)	-0.31 (0.02)
	Feeder connections density	0.03 (0.85)	-0.10 (0.47)
	Feeder connections cost	-0.17 (0.25)	-0.22 (0.11)
	Local connections density	0.43 (0.002)	0.32 (0.02)
[SI-Adol group]	Local connections cost	0.05 (0.75)	0.17 (0.22)
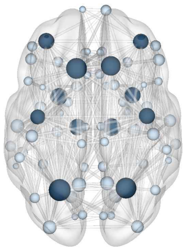	Rich club connections density	-0.34 (0.02)	-0.07 (0.63)
	Rich club connections cost	-0.32 (0.03)	-0.22 (0.12)
	Feeder connections density	0.04 (0.80)	-0.16 (0.25)
	Feeder connections cost	-0.16 (0.28)	-0.28 (0.04)
	Local connections density	0.33 (0.02)	0.22 (0.12)
[AI-Adult group]	Local connections cost	-0.09 (0.51)	0.04 (0.76)

Rich club members are defined as the top 12 highest ranking nodes on based on the degrees of the group-averaged structural networks in each AI-Adol, SI-Adol, and AI-Adult group. Network metrics of rich club organization were repeatedly compared between AI-Adol and SI-Adol groups as well as between AI-Adol and AI-Adult groups based on the information from the rich club members in each group.

Supplementary Table 3. Feeder Connections: Non-rich club regions that were connected with each rich club node

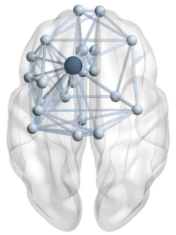	Non-rich club regions that were connected with ***'left superior frontal cortex'*** : rostral middle frontal cortex (L), caudal middle frontal cortex (L), pars opercularis (L), pars triangularis (L), medialorbitofrontal cortex (L), precentral cortex (L), paracentral cortex (L), frontal pole (L), postcentral cortex (L), rostral anterior cingulate cortex (L), caudal anterior cingulate cortex (L), posterior cingulate cortex (L), insula (L), caudate (L), pallidum (L), rostral middle frontal cortex (R), precentral cortex (R), paracentral cortex (R), frontal pole (R), rostral anterior cingulate cortex (R), caudal anterior cingulate cortex (R), pallidum (R)
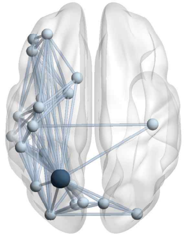	Non-rich club regions that were connected with ***'left superior parietal cortex'*** : rostral middle frontal cortex (L), pars triangularis (L), pars orbitalis (L), precentral cortex (L), inferior parietal cortex (L), supramarginal cortex (L), postcentral cortex (L), superior temporal cortex (L), middle temporal cortex (L), inferior temporal cortex (L), fusiform (L), insula (L), lateral occipital cortex (L), cuneus (L), pericalcarine cortex (L), lingual cortex (L), caudate (L), pallidum (L), postcentral cortex (R), lateral occipital cortex (R), cuneus (R)
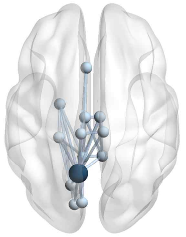	Non-rich club regions that were connected with ***'left precuneus'*** : paracentral cortex (L), caudal anterior cingulate cortex (L), posterior cingulate cortex (L), isthmus cingulate cortex (L), entorhinal cortex (L), parahippocampal cortex (L), cuneus (L), pericalcarine cortex (L), lingual cortex (L), paracentral cortex (R), posterior cingulate cortex (R), isthmus cingulate cortex (R)
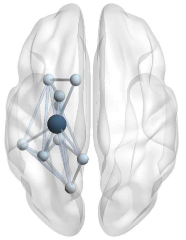	Non-rich club regions that were connected with ***'left hippocampus'*** : isthmus cingulate cortex (L), inferior temporal cortex (L), fusiform (L), entorhinal cortex (L), temporal pole (L), parahippocampal cortex (L), lingual cortex (L), amygdala (L), caudate (L)
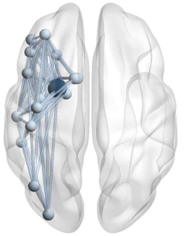	Non-rich club regions that were connected with ***'left putamen'*** : rostral middle frontal cortex (L), pars opercularis (L), pars triangularis (L), pars orbitalis (L), lateral orbitofrontal cortex (L), precentral cortex (L), inferior parietal cortex (L), postcentral cortex (L), superior temporal cortex (L), inferior temporal cortex (L), temporal pole (L), insula (L), lateral occipital cortex (L), nucleus accumbens (L), caudate (L), pallidum (L)
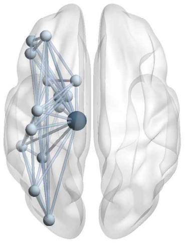	Non-rich club regions that were connected with ***'left thalamus'*** : rostral middle frontal cortex (L), pars triangularis (L), pars orbitalis (L), precentral cortex (L), inferior parietal cortex (L), postcentral cortex (L), inferior temporal cortex (L), fusiform (L), entorhinal cortex (L), temporal pole (L), lateral occipital cortex (L), amygdala (L), caudate (L), pallidum (L)

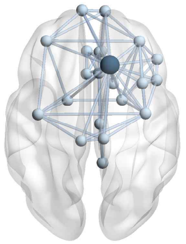	Non-rich club regions that were connected with ***'right superior frontal cortex'*** : rostral middle frontal cortex (R), caudal middle frontal cortex (R), pars opercularis (R), pars triangularis (R), medialorbitofrontal cortex (R), precentral cortex (R), paracentral cortex (R), frontal pole (R), rostral anterior cingulate cortex (R), caudal anterior cingulate cortex (R), posterior cingulate cortex (R), isthmus cingulate cortex (R), caudate (R), pallidum (R), rostral middle frontal cortex (L), precentral cortex (L), paracentral cortex (L), frontal pole (L), caudal anterior cingulate cortex (L), pallidum (L)
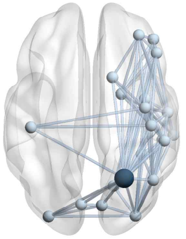	Non-rich club regions that were connected with ***'right superior parietal cortex'*** : rostral middle frontal cortex (R), pars triangularis (R), pars orbitalis (R), precentral cortex (R), inferior parietal cortex (R), supramarginal cortex (R), postcentral cortex (R), superior temporal cortex (R), middle temporal cortex (R), inferior temporal cortex (R), fusiform (R), insula (R), lateral occipital cortex (R), cuneus (R), lingual cortex (R), caudate (R), pallidum (R), postcentral cortex (L), lateral occipital cortex (L), cuneus (L)
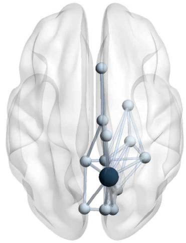	Non-rich club regions that were connected with ***'right precuneus'*** : paracentral cortex (R), caudal anterior cingulate cortex (R), posterior cingulate cortex (R), isthmus cingulate cortex (R), fusiform cortex (R), entorhinal cortex (R), parahippocampal cortex (R), cuneus (R), pericalcarine cortex (R), lingual cortex (R), isthmus cingulate cortex (L), cuneus (L)
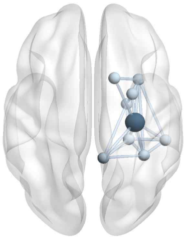	Non-rich club regions that were connected with ***'right hippocampus'*** : isthmus cingulate cortex (R), inferior temporal cortex (R), fusiform (R), entorhinal cortex (R), temporal pole (R), parahippocampal cortex (R), amygdala (R), caudate (R)
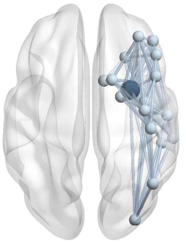	Non-rich club regions that were connected with ***'right putamen'*** : rostral middle frontal cortex (R), pars opercularis (R), pars triangularis (R), pars orbitalis (R), lateral orbitofrontal cortex (R), precentral cortex (R), inferior parietal cortex (R), postcentral cortex (R), superior temporal cortex (R), inferior temporal cortex (R), temporal pole (R), insula (R), lateral occipital cortex (R), nucleus accumbens (R), caudate (R), pallidum (R)
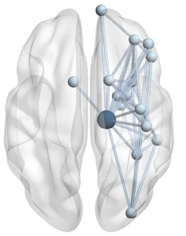	Non-rich club regions that were connected with ***'right thalamus'*** : rostral middle frontal cortex (R), pars triangularis (R), pars orbitalis (R), precentral cortex (R), frontal pole (R), postcentral cortex (R), inferior temporal cortex (R), fusiform (R), entorhinal cortex (R), temporal pole (R), lateral occipital cortex (R), amygdala (R), caudate (R), pallidum (R), caudate (L)

Supplementary Table 4. Names and abbreviations for cortical and subcortical nodes used in this study

Node name	Abbreviation	Node name	Abbreviation
Superior frontal cortex	SFC	Rostral anterior cingulate cortex	rACC
Superior parietal cortex	SPC	Caudal anterior cingulate cortex	cACC
Precuneus	preCun	Posterior cingulate cortex	PCC
Hippocampus	Hip	Isthmus cingulate cortex	ICC
Putamen	Put	Superior temporal cortex	STC
Thalamus	Thal	Middle temporal cortex	MTC
Rostral middle frontal cortex	MFC	Inferior temporal cortex	ITC
Caudal middle frontal cortex	cMFC	Fusiform	Fus
Pars opercularis	IFCa	Transverse temporal cortex	TTC
Pars triangularis	IFCb	Entorhinal cortex	Ento
Pars orbitalis	IFCc	Temporal pole	TPol
Lateral orbitofrontal cortex	OFC	Parahippocampal cortex	paraHip
Medial orbitofrontal cortex	mOFC	Banks of the superior temporal sulcus	Bank
Precentral cortex	preCen	Insula	Ins
Paracentral cortex	paraCen	Lateral occipital cortex	LOC
Frontal pole	FPol	Cuneus	Cun
Inferior parietal cortex	IPC	Pericalcarine	periCal
Supramarginal cortex	supM	Lingual cortex	Lin
Postcentral cortex	postCen		

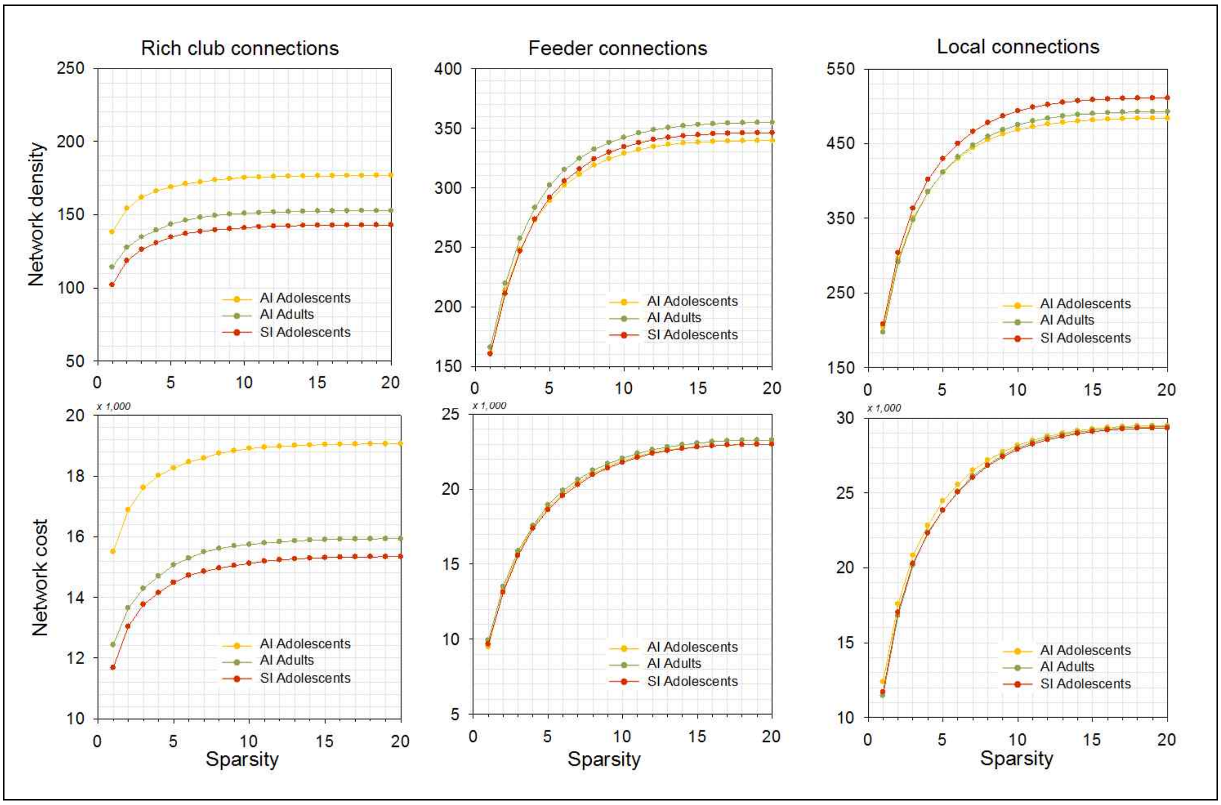

Supplementary Figure 1. Rich club organization at different sparsity for the AI-Adol (yellow), SI-Adol (red), and AI-Adult (green) groups. The area under the curve (AUC) of each network metric was computed across the range of sparsity thresholds to examine the between-group differences in network metrics.

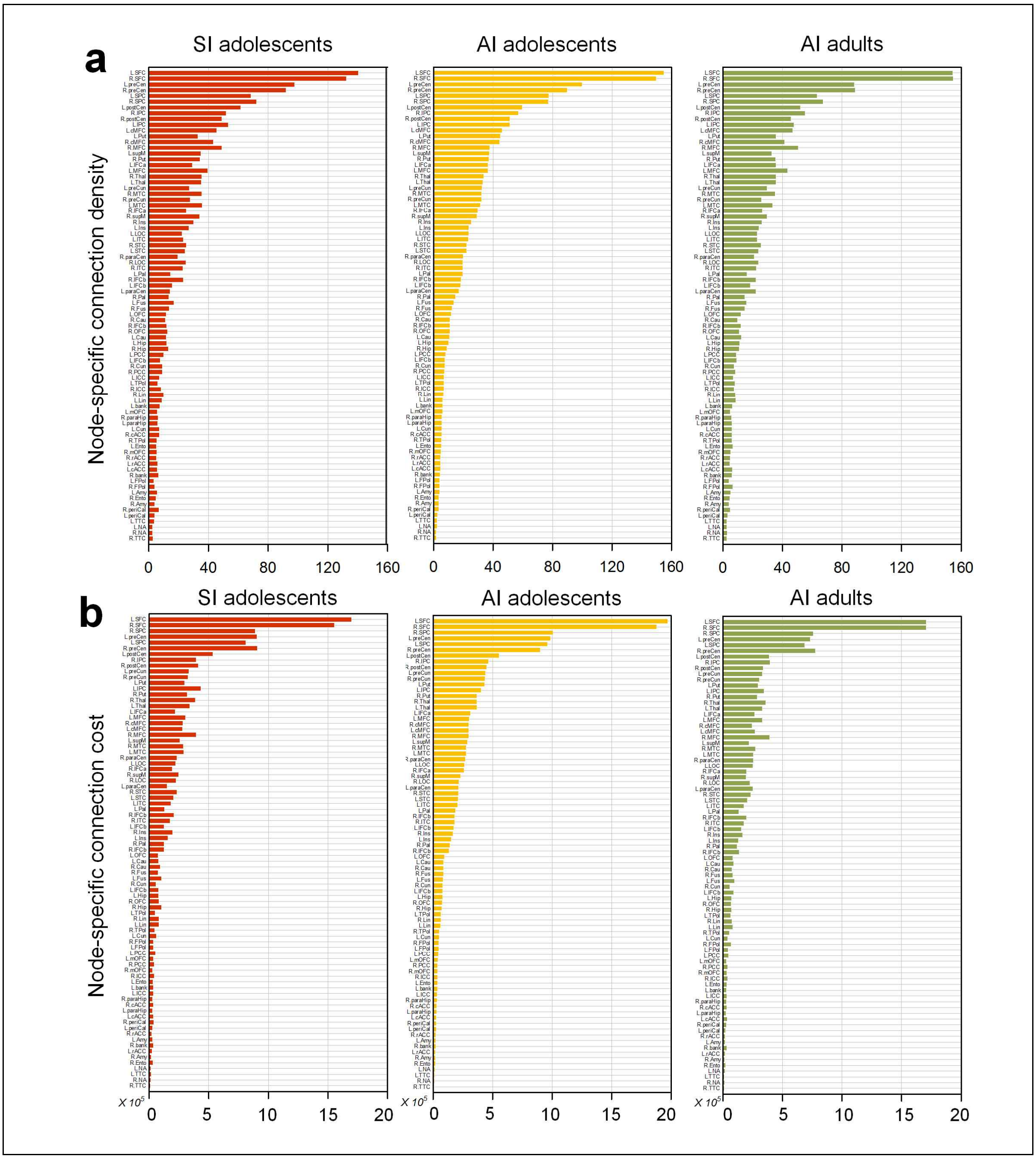

Supplementary Figure 2. Node-specific connection density (a) and cost (b) in the SI-Adol (red), AI-Adol (yellow), and AI-Adult groups (green). The nodes were ranked in descending order according to their mean connection density (a) and cost (b) across subjects within the AI-Adol group. A broadly similar pattern of ranks in node-specific connection density and cost was observed among the AI-Adol, SI-Adol, and AI-Adult groups. Please see Supplementary Table 4 for abbreviations.

References

1. Achard, S., & Bullmore, E. Efficiency and cost of economical brain functional networks. *PLoS Comput. Biol.* **3**, e17 (2007).

2. Zhang, J. *et al*. Disrupted brain connectivity networks in drug-naive, first-episode major depressive disorder. *Biol. Psychiat.* **70**, 334-342 (2011).

3. Wen, W. *et al*. Discrete neuroanatomical networks are associated with specific cognitive abilities in old age. *J. Neurosci.* **31**, 1204-1212 (2011).
